# Feeding Asian honeybee queens with European honeybee royal jelly alters body color and expression of related coding and non-coding RNAs

**DOI:** 10.3389/fphys.2023.1073625

**Published:** 2023-01-26

**Authors:** Amal Abdelmawla, Chen Yang, Xin Li, Mang Li, Chang Long Li, Yi Bo Liu, Xu Jiang He, Zhi Jiang Zeng

**Affiliations:** ^1^ Honeybee Research Institute, Jiangxi Agricultural University, Nanchang, China; ^2^ Faculty of Agriculture, Fayoum University, Fayoum, Egypt; ^3^ Jiangxi Key Laboratory of Honeybee Biology and Bee Keeping, Nanchang, Jiangxi, China

**Keywords:** honeybees, nutritional crossbreed, body color alteration, gene expression, non-coding RNA expression

## Abstract

**Background and aims:** The Asian honeybee (*Apis cerana*) and the European honeybee (*Apis mellifera*) are reproductively isolated. Previous studies reported that exchanging the larval food between the two species, known as nutritional crossbreeding, resulted in obvious changes in morphology, physiology and behavior. This study explored the molecular mechanisms underlying the honeybee nutritional crossbreeding.

**Methods:** This study used full nutritional crossbreeding technology to rear *A. cerana* queens by feeding them with an *A. mellifera* royal jelly-based diet in an incubator. The body color and the expression of certain genes, microRNA, lncRNA, and circRNA among nutritional crossbred *A. cerana* queens (NQ), and control *A. cerana* queens (CQ) were compared. The biological functions of two target genes, *TPH1* and *KMO*, were verified using RNA interference.

**Results:** Our results showed that the NQ’s body color turned yellow compared to the black control queens. Whole transcriptome sequencing results showed that a total of 1484, 311, 92, and 169 DEGs, DElncRNAs, DEmiRNAs, and DEcircRNAs, respectively, were identified in NQ and CQ, in which seven DEGs were enriched for three key pathways (tryptophan, tyrosine, and dopamine) involved in melanin synthesis. Interestingly, eight DElncRNAs and three DEmiRNAs were enriched into the key pathways regulating the above key DEGs. No circRNAs were enriched into these key pathways. Knocking down two key genes (*KMO* and *TPH1*) resulted in altered body color, suggesting that feeding NQ’s an RNAi-based diet significantly downregulated the expression of *TPH1* and *KMO* in 4-day-old larvae, which confirmed the function of key DEGs in the regulation of honeybee body color.

**Conclusion:** These findings reveal that the larval diets from *A. mellifera* could change the body color of *A. cerana*, perhaps by altering the expression of non-coding RNAs and related key genes. This study serves as a model of epigenetic regulation in insect body color induced by environmental factors.

## Introduction

The honey bee is one of the most important and beneficial insects, pollinating over 85% of global crops (Klein et al., 2007). There are nine honeybee species around the world, of which the European honeybee (*Apis mellifera*) and the Asian honeybee (*Apis cerana*) are economically the most important. Some European honeybee subspecies, for example, the Italian honeybee (*A. mellifera* ligustica), have a “yellow” body color and exhibit advantages in brood rearing, nectar collecting, and royal jelly production (Graham, 1993). On the other hand, Asian honeybees (*A. cerana*) are smaller and are primarily black body color. They are more sensitive to smell, better at exploiting sporadic nectar sources, and more suited to locating a diversity of honey plants. Asian honeybees have a significantly stronger Varroa destructor mite resistance, greater low-temperature tolerance, and decreased food consumption, compared to European bees (Chen, 2001; Zeng et al., 2006; Diao et al., 2018; Wang P. et al., 2020). Even though these two species are closely related, they have clear reproductive isolation and can’t produce filial generations. However, previous studies showed that feeding European honeybee larvae with Asian honeybee royal jelly or *vice versa*, could alter their body color, and other morphological and behavioral characteristics. This phenomenon is known as honeybee nutritional crossbreeding, and was first seen in European honeybee subspecies (Smaragdova, 1963; Rinderer et al., 1985) and is frequently reported in Asian and European honeybees. For instance, studies showed body color changes in *A. cerana* and *A. mellifera* nutritional crossbreeding (Li, 1982; Zhu, 1985; Xie et al., 2008). Xie et al. (2008) showed that nutritional crossbreeding could decrease proboscis length, wing area, and the length of the 3rd and 4th dorsal plate of the abdomen and alter the mite-defending ability of *A. mellifera* workers. It also increased the body size, birth weight, and ovariole numbers of *A. cerana* workers (Zeng et al., 2005; Chen et al., 2015). A few studies revealed that *A. cerana-A. mellifera* nutritional crossbreeding can also induce genetic changes (Xie et al., 2005a; He et al., 2010). Shi et al. (2014) showed that feeding *A. cerana* larvae with *A. mellifera* royal jelly could alter the genome-wide alternative splicing of *A. cerana*.

One commonly observed phenomenon of *A. mellifera* and *A. cerana* nutritional crossbreeding is a change in body color. In honeybees, body color is considered a heritable morphological trait used in taxonomy. However, body color changes in nutritional crossbreeding are epigenetic alterations induced by larval diets. Diet-induced body color alternation is common in many animals. For instance, feeding turtles (*Pelodiscus sinensis*) with a xanthophyll-b diet enhances the “yellowness” of their body color (Wang Z.-L. et al., 2020). Adding plant carotenoids to the diet of the Ornamental dwarf cichlid (*Microgeophagus ramirezi*) affects their color enhancement (Padowicz and Harpaz, 2007). Diet can also affect body color in the Baikal endemic amphipod (*Eulimnogammarus cyaneus*) (Saranchina et al., 2021). A recent study also confirmed that environmental factors could alter the body color of fruit flies, and this change can be passed to the next-generation (Golic et al., 1998).

Honeybee royal jelly is secreted by the hypopharyngeal and mandibular glands of nurse bees and contains major-royal-jelly-family proteins (MRJPs), amino acids, sugars, vitamins, organic acids as well as DNA and RNA (Yeung and Argüelles, 2019). Previous studies have shown differences between *A. cerana* and *A. mellifera* royal jelly with regard to 10-hydroxy-α-decenoic acid, total carbohydrate, proteins, amino acids, DNA, and microRNA components (Zeng et al., 2006; Zou et al., 2007; Oldroyd and Wongsiri, 2009; Yu et al., 2010; Liu et al., 2014). The royal jelly is powerful and important for honeybee caste differentiation so that larvae with the same genetic background fed with royal jelly develop into queens compared to larvae fed with worker jelly who develop into workers (Winston, 1991). Notably, Shi et al. (2012) reported that there are 23 microRNAs (miRNAs) specific to *A. mellifera* royal jelly, two miRNAs specific to *A. cerana* royal jelly, and 46 miRNAs that are significantly differentially expressed in both types of royal jelly. However, it is still unclear how the differences between *A. cerana* and *A. mellifera* royal jelly affects nutritional crossbreeding and the resultant altered body color.

Melanins are commonly associated with black and brown pigmentation, and play a key role in the determination of insect body color (Sugumaran and Barek, 2016). Dopamine melanin is the most common melanin pigment found in insects, and is synthesized from tyrosine in the epidermal cells (Chen et al., 2019; Wang et al., 2022). Dopamine is converted to N-β-alanyldopamine (NBAD) and N-acetyldopamine (NADA) by NBAD synthase and arylalkylamine N-acetyltransferase (aaNAT), respectively. The synthetic pathway of NBAD requires β-alanine, which is derived from L-aspartic acid by aspartate 1-decarboxylase (ADC). In addition, NBAD can be converted to dopamine and β-alanine by NBAD hydrolase (NBADH), which catalyzes the reverse reaction of NBADS (Futahashi and Osanai-Futahashi, 2021). Sasaki et al. (2021) quantified the functional monoamines (dopamine, tyramine, octopamine, and serotonin) and their precursors found in bumble bees (*Bombus ignitus*). These monoamines are synthesized from amino acids (Arakane et al., 2016; Noh et al., 2016; Sasaki et al., 2021), where tyrosine or tryptophan are important for the determination of body color (Wang et al., 2022).

Furthermore, key pathways and related key genes involved in the synthesis of melanins are an insect’s main molecular mechanisms in body color alteration. Previous studies suggest that the expression patterns and levels of *yellow* and *ebony* genes together, determine the patterns and intensity of melanization (Wittkopp et al., 2002). Borycz et al. (2002) also explain that *ebony* and *tan*, two cuticle melanizing mutants, regulate the conjugation (*ebony*) of β-alanine to dopamine or hydrolysis (*tan*) of the β-alanyl conjugate to liberate dopamine in fruit flies (*D. melanogaster*). In these fruit flies, two key genes namely *tyrosine hydroxylase* (*TH*) and *dopa decarboxylase* (*DDC*) participate in the conversion of tyrosine to dopamine (Tang, 2009), contributing to the formation of body color. In butterflies (*Bicyclus anynana*), deletion of the *yellow* and *DDC* melanin genes alters both the body color and the wing morphology (Matsuoka and Monteiro, 2018). In the pea aphid (*Acyrthosiphon pisum*), the biosynthetic pathways of amino acids (phenylalanine, tyrosine, and dopamine pathways) played a key role in cuticle formation during parthenogenetic development (Rabatel et al., 2013).

In honeybees, knocking out the *yellow-y* gene decreased the amount of black pigment in the cuticle of mosaic workers of *A. mellifera.* The expression of *Amyellow-y* and *aaNATA* in mutant drones, which have a dramatic body pigmentation defect, was lower than in wild-type drones, whereas the expression of *laccase2* was significantly upregulated (Nie et al., 2021). Additionally, seven genes involved in the biosynthesis of melanin and sclerotizing compounds are upregulated in the pharate-adults and newly-emerged bees. The gene *dopamine N-acetyltransferase* (*Dat*) and *ebony* might also contribute to body color changes in the eusocial *Frieseomelitta varia* and the solitary *Centris analis*, where the *TH* gene also showed a significantly higher expression level in the early developmental phase (Ne) than in the later phase of the three bee species (Falcon et al., 2019). By using RNA-Seq, 17 genes in *A. mellifera*, and 18 genes in *F. varia* and *C. analis*, including the *cardinal, scarlet, brown, vermillion, light, sepia*, and *henna* genes, were predicted to contribute to the formation of the adult cuticle (Falcon et al., 2019). Furthermore, Elias-Neto et al. (2010) identified an *Amlac2* gene that encodes for a laccase2 in *A. mellifera*. The *Amlac2* is highly expressed in the adult integument of pharate adults and is present before cuticle coloring and sclerotization intensify. The exoskeleton’s structural defects driven by post-transcriptional *Amlac2* gene knockdown had a significant impact on adult eclosion. Together, these findings show that ecdysteroids regulate *Amlac2* expression and are essential for the development of the adult honey bee exoskeleton. However, the molecular mechanisms of body color alteration in honeybees remain unclear, especially the color alteration induced by *A. cerana* and *A. mellifera* nutritional crossbreeding which achieves body color alternation epigenetically.

In this study, we artificially reared *A. cerana* queens in an incubator using an *A. mellifera* royal jelly based diet to get fully nutritionally crossbred queens (NQs). We also compared gene expression between NQs and control *A. cerana* queens (CQs), to identify the body color controlling pathways and related genes. Since the smallRNAs in *A. cerana* and *A. mellifera* royal jelly are different, and research has shown that the body color of fruit flies can be regulated by microRNA (Kennell et al., 2012), we hypothesized that small RNAs play a key role in *A. cerana-A. mellifera* nutritional crossbreeding related color changes. Here we compared both coding and non-coding RNA expression between NQ and CQ, to identify the body color controlling pathways, related key genes, and non-coding RNAs. This study allowed us to explore the epigenetic mechanism of nutritional crossbreeding in two honeybee species and serves as a model of epigenetic modification and phenotypic plasticity induced by nutritional diets.

## Materials and methods

### Insects

Six healthy Asian honeybee colonies (*A. cerana*) were used as larvae suppliers. Each colony had a mated queen and 12,000 worker bees. Three strong European honeybee (*A. mellifera*) colonies, each with a mated queen and more than 30,000 worker bees, were used to produce fresh royal jelly. All colonies were kept in the Honeybee Research Institute, Jiangxi Agricultural University, China.

### Diet format

Fresh 2nd-day *A. mellifera* royal jelly (RJ) was produced according to (Wu et al., 2015). The diet formula was as follows: fresh *A. mellifera* RJ 53%, 6% D-glucose (purity: analytical reagent, Xilong Scientific, China), 6% D-fructose (purity: ≧99%, Solarbio life sciences, China), 1% yeast extract (Lot: 2194133, Oxoid Ltd., United Kingdom), and 34% distilled water.

### Queen rearing

Six healthy *A. cerana* and three *A. mellifera* colonies were used for egg-laying, and the queens were caged in an empty comb for 6 h according to (Zhu et al., 2017). The queens were released and combs with eggs were placed into a queenless area of the hive. Similarly, half of the newly hatched *A. cerana* larvae (6 h) were transplanted into 24-cell tissue culture plates and then incubated at 35°C and 95% ± 3% relative humidity (RH). Each cell with one *A. cerana* larva was primed with 200–400 µL food formula (increased daily according to the larval age). For pupation, 6-day-old larvae were transferred to 6-cell tissue culture plates lined with a piece of Kimwipe and kept in an incubator at 35°C and 80% RH. Fully developed queens with a body size exceeding 220 mg, notches in their mandibles and at least 16 days of development were sampled. The rest of the hatched *A. cerana* larvae were transplanted into wax queen cells to rear as natural queens in their naive colonies for the control group. The *A. cerana* queens artificially reared by feeding with an *A. cerana* royal jelly (AcRJ) diets was considered as the control group, however, the limitation of AcRJ production could not provide enough AcRJ for artificially rearing. Additionally, it is also extremely difficult to artificially rear *A. cerana* queens based on the AcRJ diet in an incubator. Consequently, we used the natural *A. cerana* queens as the control group. After emergence, 12 fully developed NQ queens and 12 CQ queens were sampled for body color measuring and whole transcriptome sequencing. The body color was measured using Ruttner’s color scale (Ruttner, 1988). This scale classifies the amount of light pigmentation present based on an empirical series of pigmentation patterns; It is comparable with tergites, and ranges from class 0 (completely dark) to class 9 (completely yellow). The data of color scales from NQs (*n* = 4) and CQs (*n* = 4) were compared by independent-sample *t*-test (2-tailed) using the SPSS package (v25), and *p* < 0.05 was considered as significant difference.

### RNA extraction, library preparation, and whole transcriptome sequencing

All collected samples (NQ and CQ queens) were immediately stored in liquid nitrogen for future RNA extraction. Three queens from the same treatment were fixed together as one sample, and each treatment had three biological replicates. The total RNA of each sample was extracted using TRIzol reagent (Invitrogen, United States). The quality and quantity of the RNA were assessed using the RNA Nano 6000 Assay Kit of the Bioanalyser 2100 system (Agilent Technologies, CA, United States) (Liu et al., 2019). 3 μg of RNA per sample was used as input material for RNA sample preparations. After removing the ribosomal RNA and rRNA-free residue, we used rRNA-depleted RNA to construct sequencing libraries using the NEBNext^®^ Ultra™ Directional RNA Library Prep Kit from Illumina^®^ (NEB, United States). First-strand cDNA was synthesized using a random hexamer primer and Reverse Transcriptase. Second-strand cDNA synthesis was subsequently completed using DNA polymerase I and RNase H. Any remaining overhangs were converted into blunt ends *via* exonuclease/polymerase activity. After adenylation of the 3’ ends of the DNA fragments, the NEBNext Adaptor with hairpin loop structure was ligated to prepare for hybridization.

The clustering of the index-coded samples was performed on the act Cluster Generation System using TruSeq PE Cluster Kitv3-cBot-HS (Illumia) according to the manufacturer’s instructions. After cluster generation, the library preparations were sequenced on an Illumina Hiseq platform and paired-end reads were generated.

### Analysis of raw data

Raw data (raw reads) in fastq format were firstly processed in-house per scripts. In this step, clean reads were obtained by removing reads containing adapters, or Ploy-N segments and reads of low quality from the raw data. At the same time, Q20, Q30, GC-content, and sequence duplication levels of the clean data were calculated. All the downstream analyses were based on clean data with high quality. The clean reads from each sample were sequence-aligned with the designated reference *A. cerana* genome (ASM1110058v1), where the efficiency of the alignment varied from 99.69% to 99.89%. The correlation values of the three biological replicates for each sample calculated for lncRNA, miRNA, sRNA, and circRNA are presented in Supplementary Table S3.

Hereafter, sequences were aligned with the specified reference genome to obtain mapped data. Based on the mapped data, the quality of sequencing libraries was evaluated with the insert length test and the randomness test. The transcriptome was assembled using StringTie (Kovaka et al., 2019), based on the reads mapped to the reference genome. RNA-Seq was used for quality control of the clean reads from lncRNA, miRNA, sRNA, and circRNA. The assembled transcripts were annotated using the Cuffcompare program (Pertea and Pertea, 2020). Unknown transcripts were used to screen for putative lncRNAs. The different types of lncRNAs including lncRNA, intronic lncRNA, anti-sense lncRNA, and sense lncRNA were selected using Cuffcompare (Trapnell et al., 2010). Bowtie (version v1.0.0) is a short sequence comparison software (Langmead et al., 2009), especially suitable for high-throughput sequencing. This software uses the Silver database, GtRNAdb database, Rfam database, and Repbase database for sequence comparison and filtering of ribosomal RNA (rRNA), transport RNA (tRNA), Intranuclear small RNA (snRNA), nucleolar small RNA (snoRNA) and other ncRNAs and repeat sequences to get unannotated reads containing miRNAs. The percentage of Q30 base in each sample (Table 1) is evidence that the high-quality RNA-Seq data acquired could be used for further analysis.

**TABLE 1:** The statistics of coding and non-coding RNAs.

Samples	LncRNA and mRNA			circRNA			sRNA		
	Total reads	Mapped reads	Q30 (%)	Total reads	Mapped reads	Q30 (%)	Total reads	Mapped reads	Q30 (%)
Ac-NQ-1	118,764,536	90,975,067 (76.60%)	95.09	118,764,536	118,715,250 (99.96%)	98.47%	30,413,276	22,484,016 (73.93%)	96.93
Ac-NQ-2	109,272,510	69,090,736 (63.23%)	95.13	109,272,510	109,145,690 (99.88%)	98.46%	23,026,242	15,309,482 (66.49%)	96.96
Ac-NQ-3	113,155,188	73,727,115 (65.16%)	95.33	113,155,188	112,900,024 (99.77%)	98.49%	18,722,227	12,006,471 (64.13%)	96.62
Ac-CQ-1	113,048,850	86,497,271 (76.51%)	93.27	113,048,850	112,997,938 (99.95%)	97.64%	26,516,925	18,886,608 (71.22%)	95.9
Ac-CQ-2	131,028,148	94,865,818 (72.40%)	94.11	131,028,148	130,761,452 (99.80%)	97.99%	16,800,308	10,104,645 (60.15%)	96.16
Ac-CQ-3	146,759,188	131,831,300 (89.83%)	93.55	146,759,188	146,566,742 (99.87%)	97.70%	17,412,842	12,242,110 (70.31%)	97.59

Note: Total clean reads: The number of clean reads, as single-ended; Mapped reads: the number of reads on the reference genome and the percentage of them in clean reads. Q30 (%): Percentage of bases with a clean data mass value greater than Q30. In sRNA, Total reads: the number of uncommented reads used to compare with the reference genome; Mapped reads: clean reads to the reference genome.

### Correlation analysis

The correlation values of the three biological replicates for each sample calculated for lncRNA, mRNA, sRNA, and circRNA are presented in Supplementary Table S1. We excluded the NQ1 and CQ3 samples from further analysis due to their low Pearson’s correlation coefficient.

### Gene and non-coding RNA expression analysis

StringTie (v1.3.1) was used to calculate FPKMs (fragments per kilo-base of exon per million fragments mapped calculated based on the length of the fragments and reads count mapped to this fragment) of both lncRNAs and coding genes in each sample. Gene FPKMs were computed by summing the FPKMs of transcripts in each gene group. Differential expression analysis of two treatments based on read counts was performed using the DESeq2 R package (v1.10.1) (Sun et al., 2013). DESeq2 provides statistical routines for determining differential expression in digital gene expression data using a model based on the negative binomial distribution. The resulting *p-*values were adjusted using Benjamini and Hochberg’s approach for controlling the false discovery rate (FDR). Genes with an adjusted *p*-value < 0.05 and the absolute value of log2 (fold change) > 1 found by DESeq, were labelled as “differentially expressed.” Before differential gene expression analysis, the read counts were adjusted through one scaling normalized factor for each sequenced library using the edgeR program package (Robinson et al., 2010) through one scaling normalized factor. Differential expression analysis of two samples was performed using the EBseq (2010) R package. The resulting FDR (false discovery rate) was adjusted using the PPDE (posterior probability of being DE). The FDR < 0.05 and |log2(Fold Change)| ≥ 1 were set as the threshold for significant differential expression. Similarly, the significantly differentially expressed miRNAs (DEmiRNAs) and circRNAs (DEcircRNAs) were identified using DESeq2 for differential expression analysis, and log2(FC)| ≥ 1.00 was used as a threshold. The FDR values < 0.05 was used as the key indicator for differential expression miRNA and circRNA screening according to previous studies (Robinson et al., 2010; Love et al., 2014).

### GO and kyoto encyclopedia of genes and genomes (KEGG) enrichment analysis

Genes were annotated to various protein and nucleotide sequence databases using BLASTX (version 2.2.28), including the Nr (NCBI non-redundant protein sequences), Pfam (Protein family), KOG/COG (Clusters of Orthologous Groups of proteins), Swiss-Prot (A manually annotated and reviewed protein sequence database) and non-redundant nucleotide sequence (Nt) databases with a cutoff E-value of 10^–5^. GO enrichment analysis of DEGs, DElncRNAs, and DEmiRNAs was implemented using the topGO R packages (*p* < 0.01 indicates significance). The top 20 GO terms were selected. The KOBAS 2.0 (Xie et al., 2011) software was used to test the statistical enrichment (Q-value < 0.05) of differentially expressed genes and non-coding RNAs in KEGG pathways.

### RNAi experiment

We investigated the function of *TPH1* and *KMO* genes in cuticle pigmentation in *A. cerana* queens, according to the methodology of Mao et al. (2015). One-day-old worker larvae were fed with a semi-artificial diet in a petri dish, and were incubated at 34°C and 95% ± 3% humidity. Artificially manufactured siRNA for *TPH1* (F: GCG​ACA​ACU​GGG​CCA​UUA​ATT; R: UUA​AUG​GCC​CAG​UUG​UCG​CTT) and *KMO* (F: GGU​UGU​GGU​CGA​UCA​CCA​UTT; R: AUG​GUG​AUC​GAC​CAC​AAC​CTT) were added to the semi-artificial diet, with a final concentration of 20 μg/ml. Similarly, the negative siRNA (F: UUC​UUC​GAA​CGU​GUC​ACG​UTT; R: ACG​UGA​CAC​GUU​CGG​AGA​ATT) was added to a semi-artificial diet and fed to the control group larvae. Each group had 40 biological replicates. In total, 18 larvae (each treatment had 6 larvae) fed with the above *TPH1* (TPH1-RNAi treatment), *KMO* (KMO-RNAi treatment), and control siRNA (negative control) diets were collected on day 4 for qRT-PCR validation to verify the effect of RNAi on target gene expression. Each groups had three biological replicates and each biological replicate contained two mixed larvae. Each cDNA library had four technical replicates. The rest of the larvae were reared until they were fully-developed queens and their body color was measured according to the methodology of Ruttner et al. (2000) and Ruttner (1988). Data were analyzed using One Way ANOVA in SPSS package (v25), and *p* < 0.05 was considered as significant difference also.

Total RNA of each sample was extracted using TRIzol reagent (Tiangen, Beijing). The cDNA of each sample was synthesized from the total RNA using the Primer-Script RT reagent Kit (TaKaRa) according to the manufacturer’s instructions. Each cDNA library had 4 technical replicates. The *β*-actin gene of *A. cerana* was selected as the reference gene. The primers were designed using Primerpremier 5 (version 5.0) and produced by Shanghai GenePharma Co., Ltd., (Shanghai, China) (see Supplementary Table S6). An ABI 7500 real-time PCR machine (Applied Biosystems, United States) was used for amplification. Each 10 mL reaction tube contained 5 μL TB Green Premix Ex Taq II + Probe Master Mix V 1 μL cDNA, 0.8 μL Primer Set, 0.2 μL ROX Reference Dye II and 3 μL RNase Free ddH_2_0. The qPCR experiment was performed with an initial denaturation step of 10 min at 95°C, followed by 40 cycles of 94°C for 15 s, 60°C for 40 s, and 72°C for 35 s. A cycle threshold (Ct) was calculated by determining the point at which the fluorescence exceeded a threshold limit. For the data analysis, the relative expression of these two genes (*TPH1* and *KMO*) was calculated using the 2^−ΔΔCt^ comparative Ct method and was transformed by taking their root to be normally distributed. Data were analyzed by Independent-sample *t*-test (2-tailed) using the SPSS package (v25), and *p* < 0.05 was considered as a significant difference.

## Results

### Body-color alteration in NQ queens

**FIGURE 1:**
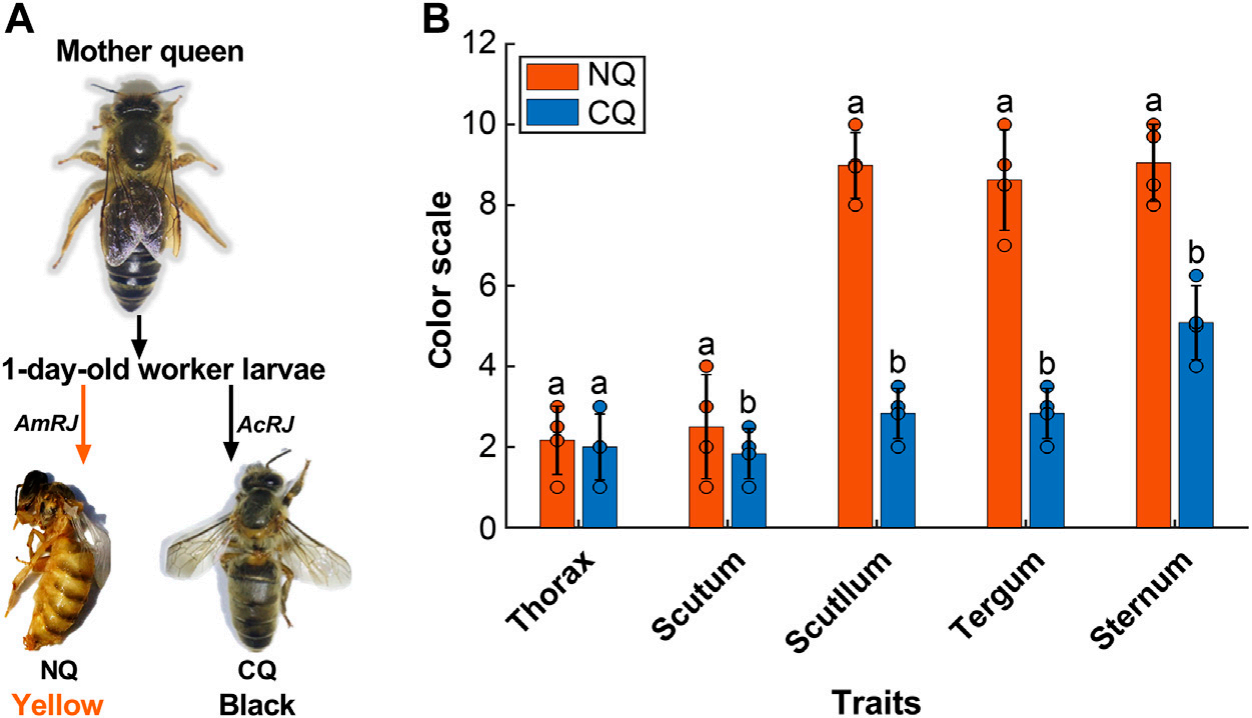
**(A)**The body color alternation in *Apis cerana-Apis mellifera* nutritional crossbreed. The 1-day worker larvae from their *A. cerana* mother queen were fed with *A. mellifera* royal jelly (*Am*RJ) based diet or *A. cerana* royal jelly (*Ac*RJ), resulted in yellow color nutritional crossbreed queens (NQ) or black color control queens (CQ) respectively. **(B)** The body color quantification had significant differences of (NQs, *n* = 4, Scutum’s M = 2.5, SD = 1.29, t = 0.85, Scutellum’s M = 8.9, SD = 0.81, *t* = 5.84, Tergum’s M = 8.5, SD = 1.25, *t* = 63.81, and Sternum’s M = 9.5, SD = 0.95, t = 15.4) compared to (CQs, *n* = 4, Scutum’s M = 1.83, SD = 0.62 *t* = 5.11, Scutellum’s M = 0.81, SD = 0.62*, t* = −16.12, Tergum’s M = 2.8, SD = 0.62 *t* = 63.81, and Sternum’s M = 5.8, SD = 0.92, *t* = 15.14) all *P* were < 0.05) based on Ruttner’s color scales. Bars present as values of Mean ± SD, the black dots on the top of each bar represent replicates. Different letters on the top of bars indicate significant difference (*p* < 0.05, Independent-sample *t*-test).

Figure 1 clearly shows that *A. cerana* queens reared on an *A. mellifera* royal jelly based diet had a significantly lighter body color compared to their black mother queens and control queens (*p* < 0.0001, Figure 1B).

### Data quality of whole transcriptome sequencing

Six whole transcriptome sequencing libraries of NQ queens and CQ queens were established. In total, 108.0 2 GB of clean reads from mRNAs and lncRNAs were obtained, resulting in 16.09 GB of clear data per sample after quality control. The percentage of Q30 base of each sample was more than 93.27% (Table 1). The same six RNA-seq library samples were used to construct non-coding RNA libraries for miRNAs and circRNAs. A total of 162.29 and 366.01 M of clean reads were obtained in miRNAs and circRNAs, respectively, and the percentage of Q30 base for each sample was over 95.90% (Table 1). All results indicate high-quality RNA-Seq data. Pearson’s correlation coefficient of mRNA in all biological replicates of each group was above 0.8, except for NQ1 and CQ3 (Supplementary Table S1). Pearson’s correlation coefficient of miRNA in all biological replicates was above 0.8 and the correlation of lncRNA and circRNA in all replicates (Supplementary Table S1).

### DEGs and differentially expressed non-coding RNAs

**FIGURE 2:**
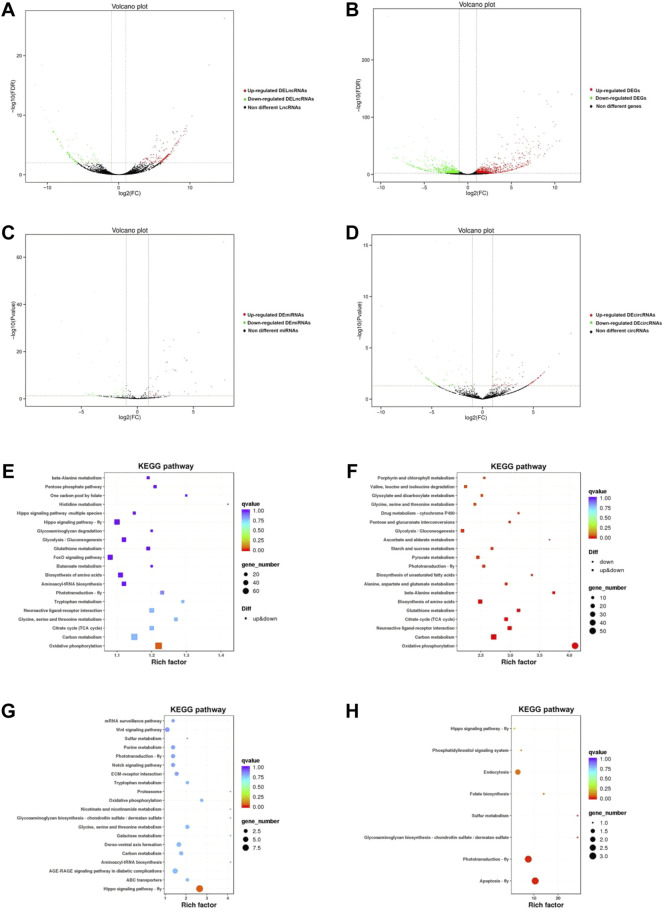
**(A)** The volcano diagram of lncRNAs between NC and NQ. The red spots represent upregulated DElncRNAs in NQ compared to CQ, whereas the green spots represent downregulated DElncRNAs; The black spots are non-different lncRNAs. **(B)** The volcano diagram of DEGs between NC and NQ. The red spots represent upregulated DEGs in NQ compared to CQ, whereas the green spots represent downregulated DEGs. lncRNAs with FDR < 0.05, |log2 (Fold change) |≧1 were identified as DElncRNAs. Same to DEGs, DEmiRNAs and DEcircRNAs. **(C)** The volcano diagram of miRNAs between NC and NQ. The red spots represent upregulated DEmiRNAs in NQ, whereas green spots represent downregulated DEmiRNAs. **(D)** The volcano diagram of circRNAs between NC and NQ. The red spots represent upregulated DEcircRNAs in NQ, whereas green spots represent downregulated DEcircRNAs. **(E–H)** are the top pathways of KEGG enrichment of DEGs, DElncRNAs, DEmiRNAs and DEcircRNAs respectively. The sizes of circles represent the number of DEGs, DElncRNAs, DEmiRNAs, and DEcircRNAs, and the colors of circles represent the *p*-values of enrichment.

In total, 1484, 311, 92, and 169 DEGs, DElncRNAs, DEmicroRNAs, and DEcircRNAs were identified between NQ and CQ respectively (Table 2; Supplementary Table S2), with 782, 209, 45, and 99 of these genes upregulated in NQ and 702, 102, 47, and 70 upregulated in CQ (Figures 2A–D). These results were constant in the sample clustering (heat map) using the full gene set (Supplementary Figure S1).

**TABLE 2:** Number of differentially expressed coding and non-coding RNAs identified from *A. cerana* artificially nutritional cross queens vs. *A. cerana* natural queens.

*NQ* vs. *CQ*	DELncRNA		DEGs		DEmiRNA		DEcricRNA	
Regulation	Up	Down	Up	Down	Up	Down	Up	Down
Number of differentially expressed genes or non-coding RNAs	209	102	782	702	45	47	99	70

### GO and KEGG enrichment

The DEGs, DElncRNAs, DEmicroRNAs, and DEcircRNAs were enriched into 53, 53, 749, and 47 GO terms respectively (Supplementary Table S3). The enriched and classification of GO terms are summarized in Supplementary Figure S2. Here the top 10 GO terms of DEGs and differentially expressed non-coding RNAs were transmembrane transport, an integral component of membrane, and NADH dehydrogenase (ubiquinone activity). KEGG enrichment analysis showed a total of DEGs, DElncRNAs, DEmiRNAs, and DEcircRNAs enriched into 123, 151, 38, and 8 KEGG pathways, respectively (Figures 2E–H; Supplementary Table S4), and one key pathway (tryptophan metabolism), formed part of the top ten KEGG pathways in DEGs (Figure 2E) and DEmiRNA (Figure 2G).

### Key KEGG pathways for body color regulation and related DEGs, DElncRNAs and DEmiRNAs

**FIGURE 3:**
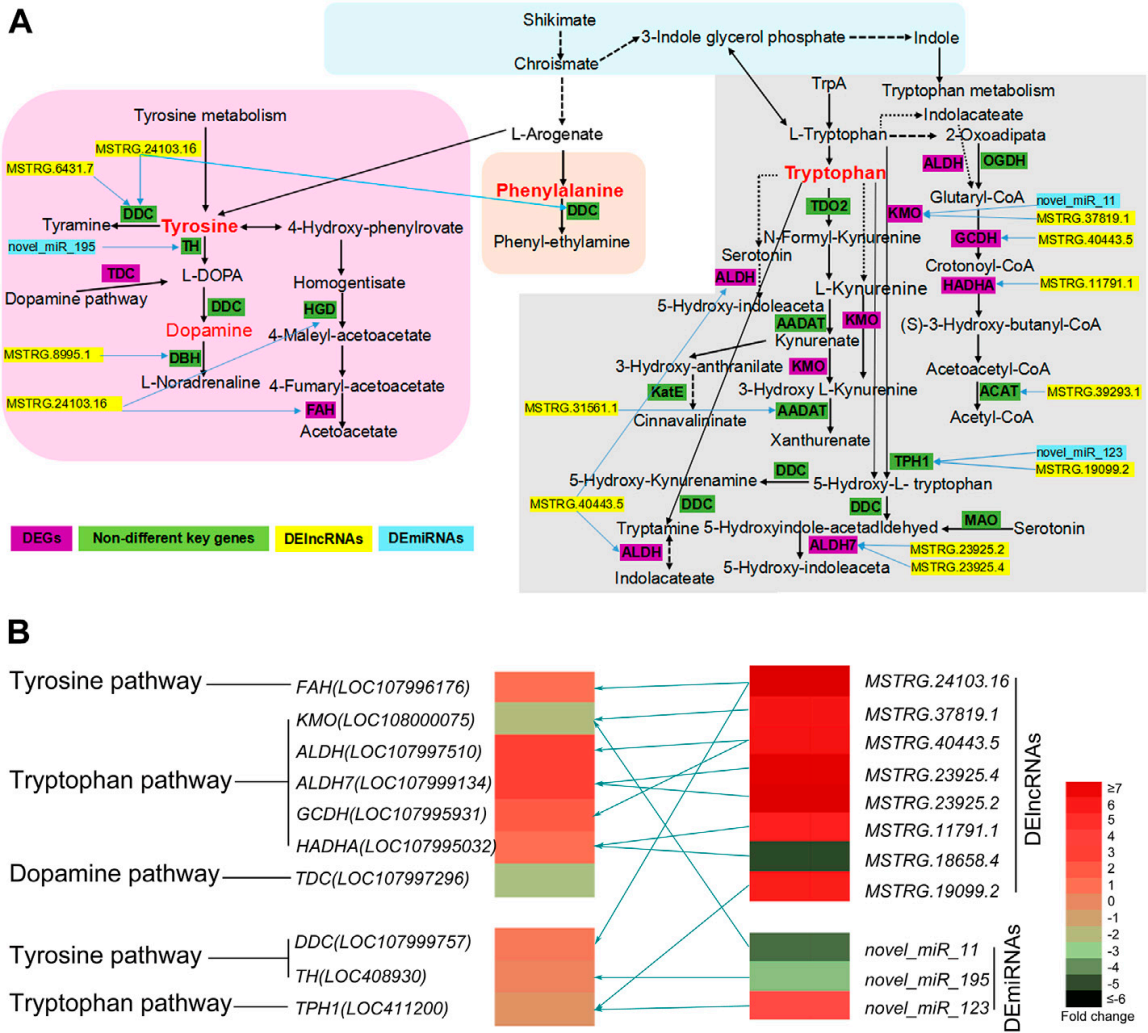
**(A)** The predicted KEGG network of honeybee body color alternation in nutritional crossbreed. This network is based on four key KEGG pathways including tyrosine, tryptophan, dopamine, and phenylalanine pathways. The key DEGs, DElncRNAs and DEmiRNAs involved in this network are also presented. Yellow bars represent DElncRNAs, sky blue bars represent DEmiRNAs, and purple bars represent DEGs and green bars represent key genes but not DEGs. Blue arrows mean DElncRNAs or DEmiRNAs involved into the regulation of related genes. **(B)** The heatmap of key DEGs, DElncRNAs and DEmiRNAs are involved into three key KEGG pathways. Different colors represent significantly differential expression of key DEGs, DElncRNAs and DEmiRNAs using their log2 (Fold change) values. Olive green arrows represent the regulatory relationship between key DEGs and non-coding RNAs (DElncRNAs and DEmiRNAs).

The phenylalanine, dopamine, tryptophan, and tyrosine pathways are the most important KEGG pathways involved in insect pigmentation (Rabatel et al., 2013; Lambrus et al., 2015; Ahmad et al., 2020; Zhang et al., 2020; Sasaki et al., 2021). More interestingly, our results showed that seven DEGs were enriched in the above three key pathways, including *ALDH, ALDH7, KMO, GCDH, HADHA, FAH,* and *TDC* (Figure 3, details see Supplementary Table S5). Three key genes (*DDC, TH* and *TPH1*) were also selected and presented in Figure 3 due to their important functions in insect body color regulation (Tang, 2009; Lambrus et al., 2015; Futahashi and Osanai-Futahashi, 2021), even though their FDR or log2(FC) values did not reach the threshold of significant difference. Moreover, a total of eight DElncRNAs and three DEmiRNAs were also enriched into these key pathways (Figure 3; Supplementary Table S5), including MSTRG.24103.16, MSTRG.37819.1, MSTRG.40443.5, MSTRG.23925.4, MSTRG.23925.2, MSTRG.11791.1, MSTRG.18658.4, MSTRG.19099.2 as well as three DEmiRNAs (novel miR195, novel miR11 and novel miR123). More interestingly, the key DEGs, DElncRNAs, and DEmiRNAs showed a regulating network (Figure 3B). One of the DEGs was regulated by multiple DElncRNAs or by both DElncRNAs and DEmiRNAs together (Figure 3B).

### RNAi effect on body color key genes

**FIGURE 4:**
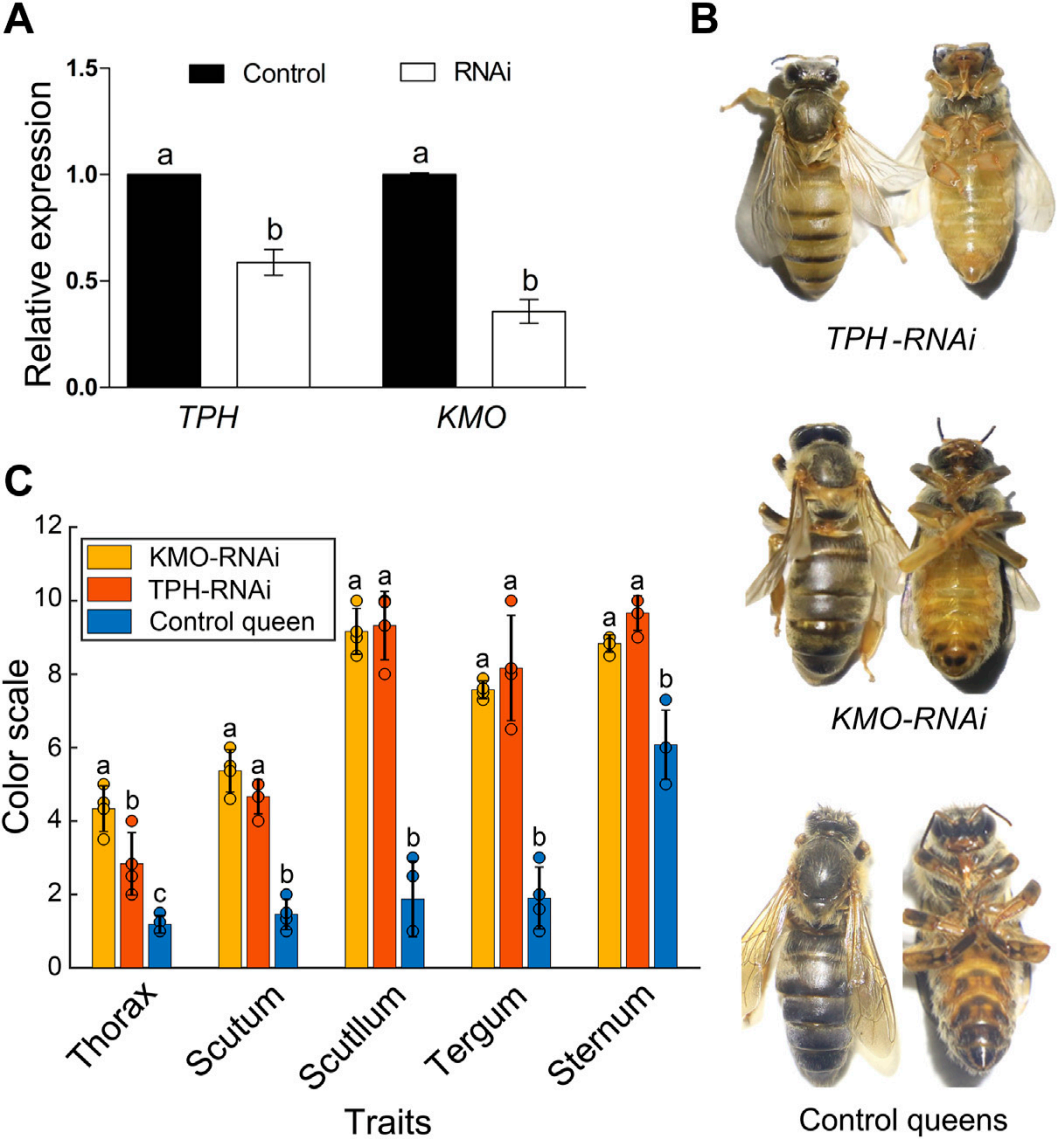
**(A)** The expression of *TPH1* and *KMO* genes in 4-day *A. cerana* queen larvae by RNAi. Bars represent mean ± SD values of relative gene expression. Different letters on the top of each bar represent the significant difference (*p* < 0.05, independent-sample *t*-test). **(B)** The body color alternation of *A. cerana* queens in RNAi experiment. The upper queens with a yellow body color are *TPH1-*RNAi group, the middle ones are *KMO* RNAi group with slight body color changes compared to control bees. The lower ones are control queens fed with negative siRNA. **(C)**The body color quantification of *KMO*-iRNA (*n* = 4), *TPH1-*iRNA (*n* = 4), and control queens (*n* = 4) based on Ruttner’s color scales. Bars are present as values of Mean ± SD. Different letters on the top of the bars indicate significant differences (*p* < 0.0001 F = 25.41 F critical = 4.25, One-Way ANOVA), the same letter indicates no significant difference, and the black dots on the top of each bar represent replicates.

The RNAi results revealed that feeding RNAi-based food significantly downregulated the expression of *TPH1-2* and *KMO* in 4-day-old larvae (*p* < 0.01, Figure 4A), resulting in a clear and significant color change in newly emerged queens (Figures 4B, C). The RNAi-treated queens exhibited a notable increase in yellow pigment and lacked black color, whereas the control queens developed normally and exhibited the normal black body pigmentation similar to their mother queens (Figure 4C; df for all traits = 2, while *p*-value < 0.0001 for Thorax, Scutum, Scutellum, and Sternum. For Thorax F value = 109, Scutum F value = 112, Scutellum F value = 289 Tergum: F value = 35.45, *p*-value = 0.0005, and Sternum F value = 103.20; Figure 4; Supplementary Figure S3). This confirms that the *TPH1* and *KMO* genes play an important role in the formation of honeybee body color and pigmentation.

## Discussion

Nutritional crossbreeding between Asian and European honey bees induces morphological, physiological, and behavioral changes in the adults (Smaragdova, 1963; Rinderer et al., 1985; Ruttner, 1988; Shi et al., 2014), which renders it an optimal model for studies on the epigenetic mechanisms of animal phenotypic plasticity. The change in body color is one of the most observed changes in honeybee nutritional crossbreeding. In this study, we artificially reared fully nutritionally crossbred *A. cerana* queens on *A. mellifera* RJ diets. Our results showed a clear change in body color from black to yellow (Figure 1). We also identified a regulation network of eight DElncRNAs and three DEmiRNAs that regulate key DEGs involved in the melanin synthesis pathways. These results revealed that non-coding RNAs presumably participate in honeybee body color alteration induced by *A. cerana*-*A. mellifera* nutritional crossbreeding by regulating the expression of related key genes. We note that the CQ is not an optimal control, since different rearing methods for producing NQs and CQs might, to same extent, influence the results of whole transcriptome sequencing. However, recently it is too difficult to artificially rear *A. cerana* queens using a diet format based on the *A. cerana* royal jelly. Therefore, the CQs produced by the same mother queen as NQs were selected as the control group.

### The effect of nutritional crossbreeding on NQ body color alteration

Many previous studies have reported a body color alteration in Asian and European honeybee nutritional crossbreeds by using partial nutritional crossbreeding methods. This involves rearing nutritionally crossbred queens in their native colonies by adding other species’ royal jelly in queen cells or rearing queens in a different honeybee species colony for 1–2 days and then returning them to their native colonies until emergence (Li, 1982; Zhu, 1985; Xie et al., 2008). The body color of the queen or worker bees in these studies was only partly altered (Xie et al., 2005a; Xie et al., 2005b; Zeng et al., 2005; He et al., 2010; He et al., 2011). By using a complete nutritional crossbreeding method that rears the *A. cerana* queens on an *A. mellifera* royal jelly-based diet, a clear color change of the whole body was observed. Figure 1 illustrates how the head, thorax, abdomen, and all six legs of the NQ queens were yellow, whereas the mother and control queens had black bodies and legs. This reflects the large-scale body color changes induced by nutritional crossbreeding. Consequently, the complete nutritional crossbreeding method firstly developed in this study demonstrates the powerful effects of nutritional crossbreeding and allows us to explore its underlying epigenetic mechanisms.

### Key pathways and genes involved in body color alteration

The formation of insect body color is linked to the synthesis of melanin, which is mainly regulated by a few key KEGG pathways and related key genes. The most important KEGG pathways involved in insect pigmentation are phenylalanine, tryptophan, dopamine, tyrosine, and tryptophan (Rabatel et al., 2013; Ahmad et al., 2020). We subsequently compared gene expression in NQs compared to CQs through whole transcriptome sequencing. Interestingly, seven DEGs were enriched into the above four key pathways (Figure 3), which have been shown to participate in the regulation of insect body colors (Eisner et al., 1997; Cole et al., 2005; Liu et al., 2020; Moraes et al., 2022). The *KMO* gene in silkworms acts as a transgenic marker, which turns the integument of the first instar larvae brown (Kobayashi et al., 2007). Therefore, these genes possibly play a key role in color alteration in NQs. Moreover, three genes (*TPH1, DDC,* and *TH*) are also key genes enriched into the tryptophan and tyrosine pathways and have been previously reported as key genes for the determination of insect body color, even though their FDR and log2 FC values did not reach the threshold of significant difference (Figure 3). Previous studies indicate that these three genes are essential for the formation of insect body color: The *TPH1* gene determines the eye pigmentation in the planarian *Schmidtea mediterranea* (Lambrus et al., 2015), while the *TH* and *DDC* genes participate in dopamine biosynthesis of bees and fruit flies such as *D. melanogaster* (Tang, 2009; Futahashi and Osanai-Futahashi, 2021). Moreover, two DElncRNAs (MSTRG.24103.16 and MSTRG.19099.2) and two DEmiRNAs (miR195 and miR123) were related to these three genes (Figure 3). Eventually, our RNAi results confirmed that knocking down two key genes (*KMO* and *TPH1*) resulted in the clear alteration of honeybee body color (Figure 4). Consequently, the effects of honeybee nutritional crossbreeding possibly influence bee body color by altering the expression of the above key genes. Here we note that the whole body of queens rather special tissues was used for whole transcriptome sequencing, which might conceal some other key genes. Further studies could verify our results using special tissues (for example: color-changed cuticle).

### Epigenetic modification in body color alteration of NQ

Body color is the most distinguishing and conservative morphological trait of honeybees, and have been used as a selection parameter and diagnostic character in breeding and taxonomy. The pattern of light (yellow, orange) and dark (black, brown) color differs between species as well as between the casts of the same colony (Woyke, 1997; Tilahun et al., 2016). However, in the present study, the body-color alteration in honeybees induced by nutritional crossbreeding is an epigenetic phenomenon, since this alteration was based on exchanged larval diets. Nutrients can reverse or change epigenetic phenomena such as DNA methylation and histone modification in insects, altering the expression of critical genes related to development (Choi and Friso, 2010), phenotype, and body color regulation (Golic et al., 1998; Saranchina et al., 2021). The royal jelly of honeybees is a powerful food source that determines queen-worker caste differentiation (Winston, 1991; Wojciechowski et al., 2018; Slater et al., 2020) through epigenetic modifications (Maleszka, 2008; He et al., 2017). Indeed, our results showed that thousands of non-coding RNAs were significantly differentially expressed between NQ and CQ queens (Figure 2; Table 2). Here, eight DElncRNAs and three DEmiRNAs were highly related to the gene expression regulation of key genes that are vital to body color determination (Figure 3), although no circRNAs were involved. Our results are supported by many fruit fly studies showing that miRNAs regulate insect body color (Kennell et al., 2012; Bejarano and Lai, 2021). As one of the most powerful epigenetic modifications, non-coding RNAs such as lncRNAs, circRNAs, and miRNAs play a vital role in animal phenotypic plasticity by regulating gene expression (Shi et al., 2014; Legeai and Derrien, 2015; Zhu et al., 2017; Wen et al., 2020; Choudhary et al., 2021). Such epigenetic mechanism possibly also applies in *A. cerana*-*A. mellifera* nutritional crossbreeding. Therefore, it is believed that non-coding RNAs act as a vital epigenetic modification underlying *A. cerana-A. mellifera* nutritional crossbreeding, resulting in whole-body color alteration by regulating key pathways and related key genes. Shi et al. (2012) showed that dozens of miRNAs differ between *A. cerana* and *A. mellifera* royal jellies but differences were not detected in this study, perhaps due to the short actuation duration of miRNAs that do not persist in adult queens. Exactly which key components of honeybee royal jelly affect the expression of non-coding RNAs still requires further investigation.

In summary, the study explored the epigenetic mechanisms underlying nutritional crossbreeding of two honeybee species and revealed that lncRNAs, and, miRNAs contribute to body color alteration in NQs by regulating the expression of key genes and pathways that are related to melanin synthesis. This study not only demonstrated an epigenetic mechanism underlying honeybee nutritional crossbreeding but also serves as a model for studies on the epigenetic mechanisms of animal phenotypic plasticity induced by environmental factors.

## Data Availability

The datasets presented in this study can be found in online repositories. The names of the repository/repositories and accession number(s) can be found in the article/Supplementary Material.

## Supplementary material

SUPPLEMENTARY FIGURE S1 The heat map of expression profiles of mRNAs, lncRNAs, circRNAs and sRNAs in NQs and CQs. The heat map of expression profiles of lncRNAs; **(B)** the heat map of expression profiles of mRNAs; **(C)** the heat map of expression profiles of sRNAs. **(D)** the heat map of expression profiles of circRNAs. NQ and CQ are nutritional crossbreed queens and control queens, and each had two biological replicates. The horizontal coordinates represent the sample name and the clustering results of the sample, and the ordinates represent the difference genes and the clustering results of the genes. The different columns in the figure represent different samples, and the different rows represent different genes. Colors represent the gene expression levels [log10 (FPKM plus 0.000001)] of each sample.

SUPPLEMENTARY FIGURE S2 The top enriched and classification of Go terms identified from **(A)** DEcircRNAs, **(B)** DElncRNAs, **(C)** DEGs, and **(D)** DEmiRNAs. The results are summarized in three main categories: biological process, cellular component and molecular function. The lighter colored bars in Y-axis incident the percentages of coding or non-coding RNAs in a category, and the deeper colored ones indicate the present ages of differentially expressed coding or non-coding RNAs.

SUPPLEMENTARY FIGURE S3 The body color of full developed queens from *TPH1, KMO* and control groups.

SUPPLEMENTARY TABLE S1
**(A)** Pearson correlation coefficient of mRNA. **(B)** correlation coefficient of lncRNA. **(C)** Pearson correlation coefficient of circRNA. **(D)** Pearson correlation coefficient of sRNA.

SUPPLEMENTARY TABLE S2 The list of DEGs, DELncRNA, DEmiRNA, DEcircRNAs.

SUPPLEMENTARY TABLE S3 GO enrichment of DEGs, DELncRNA, DEmiRNA, DEcircRNAs.

SUPPLEMENTARY TABLE S4 Enriched KEGG pathways of DEGs, DELncRNA, DEmiRNA, DEcircRNAs.

SUPPLEMENTARY TABLE S5 The lists of DEGs, DElncRNAs and DEmiRNAs and key KEGG pathways involved in body color.

SUPPLEMENTARY TABLE S6 The primers of the *β*-actin gene, TPH1 and KMO in RNAi.
