# Supplementary figures and images for: Feeding Asian honeybee queens with European honeybee royal jelly alters body color and expression of related coding and non-coding RNAs
Source: Front Physiol. 2023 Jan 26;14:1073625. doi: 10.3389/fphys.2023.1073625 (PMC9908965; doi:10.3389/fphys.2023.1073625)

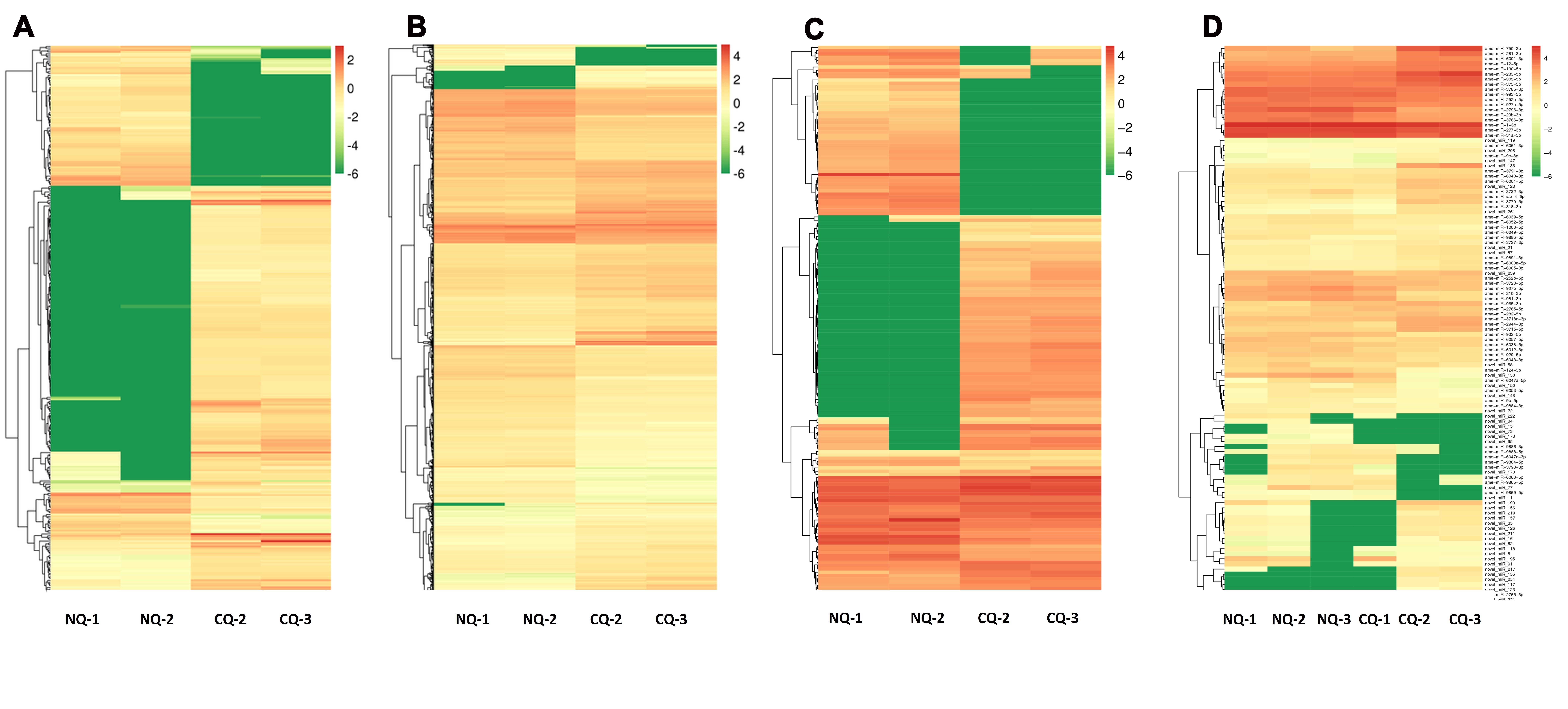

Supplement: Supplementary file 2 [file Image1.tiff]

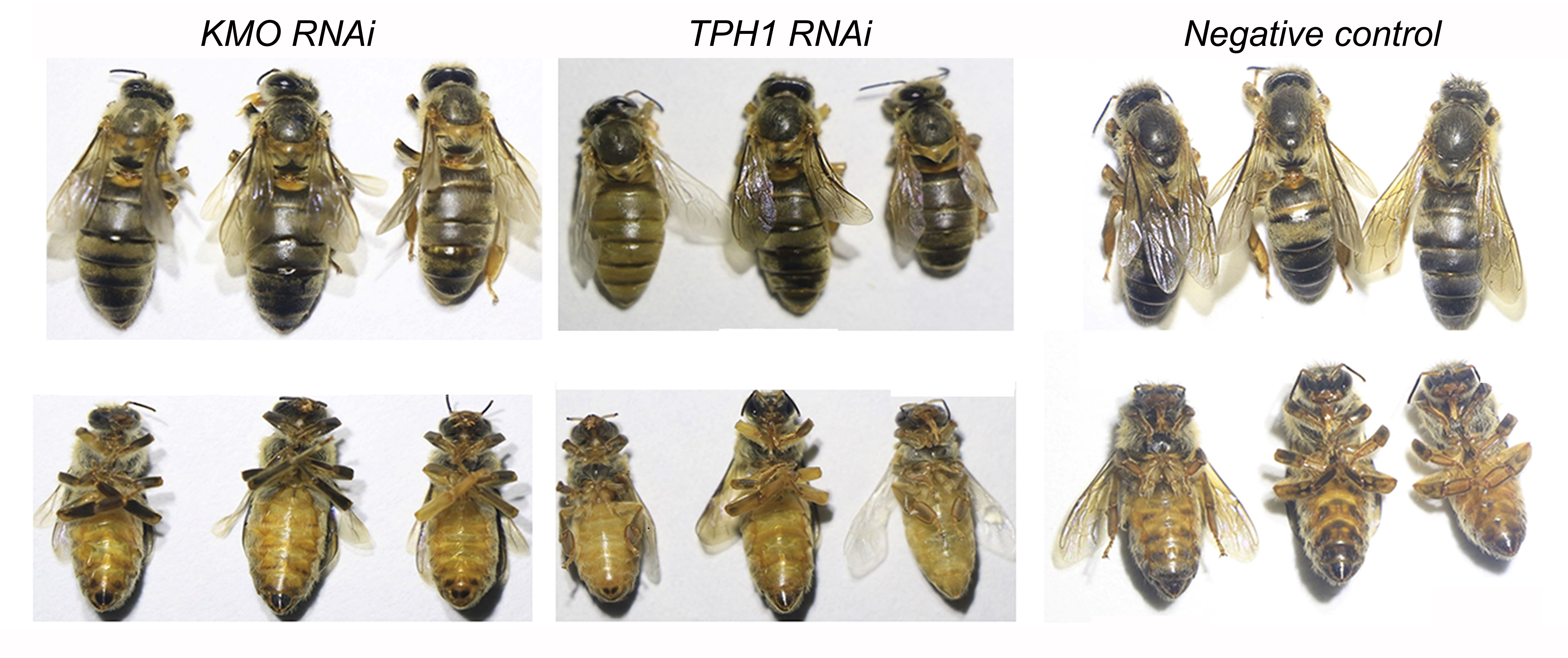

Supplement: Supplementary file 4 [file Image3.tif]
