# Supplementary material for: Feeding Asian honeybee queens with European honeybee royal jelly alters body color and expression of related coding and non-coding RNAs
Source: Front Physiol. 2023 Jan 26;14:1073625. doi: 10.3389/fphys.2023.1073625 (PMC9908965; doi:10.3389/fphys.2023.1073625)
Supplement: Supplementary file 7 [file Table6.docx]

| Gene name | Primer |
| --- | --- |
| TPH1 | F: TGGCTGGCTATCTATCACCG  R: ACCCAACAATTCGTGACAGC |
| KMO | F: CCGCGGTTCAATTGTAGTCA  R: GTCGTTGGGCAAAGCTATCA |
| β-actin | F:ATGCCAACACTGTCCTTTCTGG  R:GACCCACCAATCCATACGGA |

Table S6 The primers of the *β*-actin gene, TPH1 and KMO in RNAi.
